# Dysregulation of USP18/FTO/PYCR1 signaling network promotes bladder cancer development and progression

**DOI:** 10.18632/aging.202359

**Published:** 2021-01-10

**Authors:** Wei Song, Ke Yang, Jianjun Luo, Zhiyong Gao, Yunliang Gao

**Affiliations:** 1 Department of Urology, Hunan Provincial People’s Hospital, The First Affiliated Hospital of Hunan Normal University, Changsha 410005, Hunan Province, China; 2 Department of Urology, The Second Xiangya Hospital, Central South University, Changsha 410011, Hunan Province, China

**Keywords:** N6-methyladenosine, m6A, methylation, FTO, bladder cancer

## Abstract

N6-methyladenosine refers to a methylation of adenosine base at the 6^th^ nitrogen position, which is the dominant methylation modification in both message and non-coding RNAs. Dysregulation of RNA m6A methylation causes tumorigenesis in humans. The key N6-methyladenosine demethylase fat-mass and obesity-associated protein (FTO) is negatively correlated with the overall survival of bladder cancer patients, but the underlying mechanism remains poorly understood. In this study, we demonstrated that the post-translational deubiquitination by USP18 up-regulates the protein but not mRNA of FTO in bladder cancer tissues and cells. As a result, FTO decreased N6-methyladenosine methylation level in *PYCR1* through its demethylase enzymatic activity and stabilized *PYCR1* transcript to promote bladder cancer initiation and progression. Our work shows the importance of N6-methyladenosine RNA modification in bladder cancer development, and highlights UPS18/FTO/PYCR1 signaling network as potential therapeutic targets of bladder cancer.

## INTRODUCTION

N6-methyladenosine (m6A) is the most abundant type of modification in messenger RNAs (mRNA) and is highly conserved in both prokaryotes and eukaryotes [1]. Similar to DNA and histone methylation, RNA m6A methylation is a dynamic and reversible epi-regulation [2]. The multi-protein m6A methylase complex introduces methyl groups into RNA as a writer. Components of such methylase complex include the catalytic core enzyme of methyltransferase-like 3 (METTL3) and its molecular chaperons like METTL14 and Wilm’s tumor 1-associated protein (WTAP) [3]. On the contrary, demethylase removes methyl groups like an eraser, which reduces the cellular m6A level. The Fe (II) / α-ketoglutarate-dependent oxygenase superfamily members of Fat-mass and obesity-associated protein (FTO) [2] and ALKB homolog 5 (ALKBH5) [4] have been the major demethylases identified. These writers and erasers determine the dynamic m6A methylation profiling in RNA, which is then interpreted by reader proteins such as YT521-B homology (YTH) family members and heterogeneous nuclear ribonucleoproteins (hnRNPs) [5, 6].

Unlike epigenetic modifications of DNA and histones, which play critical roles in transcription, RNA m6A methylation mainly regulates gene expression at the post-transcriptional level. It influences RNA metabolism by participating in RNA processing, nuclear export and translation to RNA degradation [1]. Because it accounts for over 80% of all RNA methylation, m6A RNA methylation is important in regulating stress reaction, cell proliferation, stem cell differentiation, immunity, inflammation and development and so on [7]. Aberrant changes in the expression of writers and/or erasers of m6A methylation cause diseases such as diabetes, obesity, neurodegeneration and cancer [8, 9].

FTO was initially recognized as a susceptible gene for type 2 diabetes. Multiple single nucleotide polymorphisms (SNPs) in its first intron are closely related to the occurrence of obesity [10]. FTO overexpression causes obesity, whereas loss of FTO leads to growth retardation and high early mortality in rodents [11–13]. Although it has long been appreciated the carcinogenic role of FTO, the underlying mechanism via RNA m6A demethylation has been unknown until it was first proved as a common demethylase of DNA and RNA in 2011 [2]. Thereafter, FTO was shown to direct gene expression by changing the level of mRNA m6A methylation and acts as an oncogene in breast cancer [14], lung cancer [15, 16], endometrial cancer [17], acute myeloid leukemia [18, 19] as well as pancreatic cancer [20].

The role of FTO in bladder cancer (BLCA) has been ill-elucidated thus far. In this study, we demonstrated that FTO protein but not mRNA is highly expressed in BLCA tissues and cell lines due to ubiquitin Specific Peptidase 18 (USP18)-imposed post-translational deubiquitination on the N-terminal protein domain. Stabilized FTO reduced m6A methylation on pyrroline-5-carboxylate reductase 1 (*PYCR1*) and extended *PYCR1* mRNA half-life to promote BLCA cell proliferation and migration *in vitro* as well as tumor growth *in vivo*. Our study reveals a novel mechanism underlying BLCA progression through USP18/FTO/PYCR1 signaling network, and provides the potential targets for BLCA therapy.

## RESULTS

### Increased FTO protein is negatively associated with the overall survival of BLCA patients

**Figure 1:**
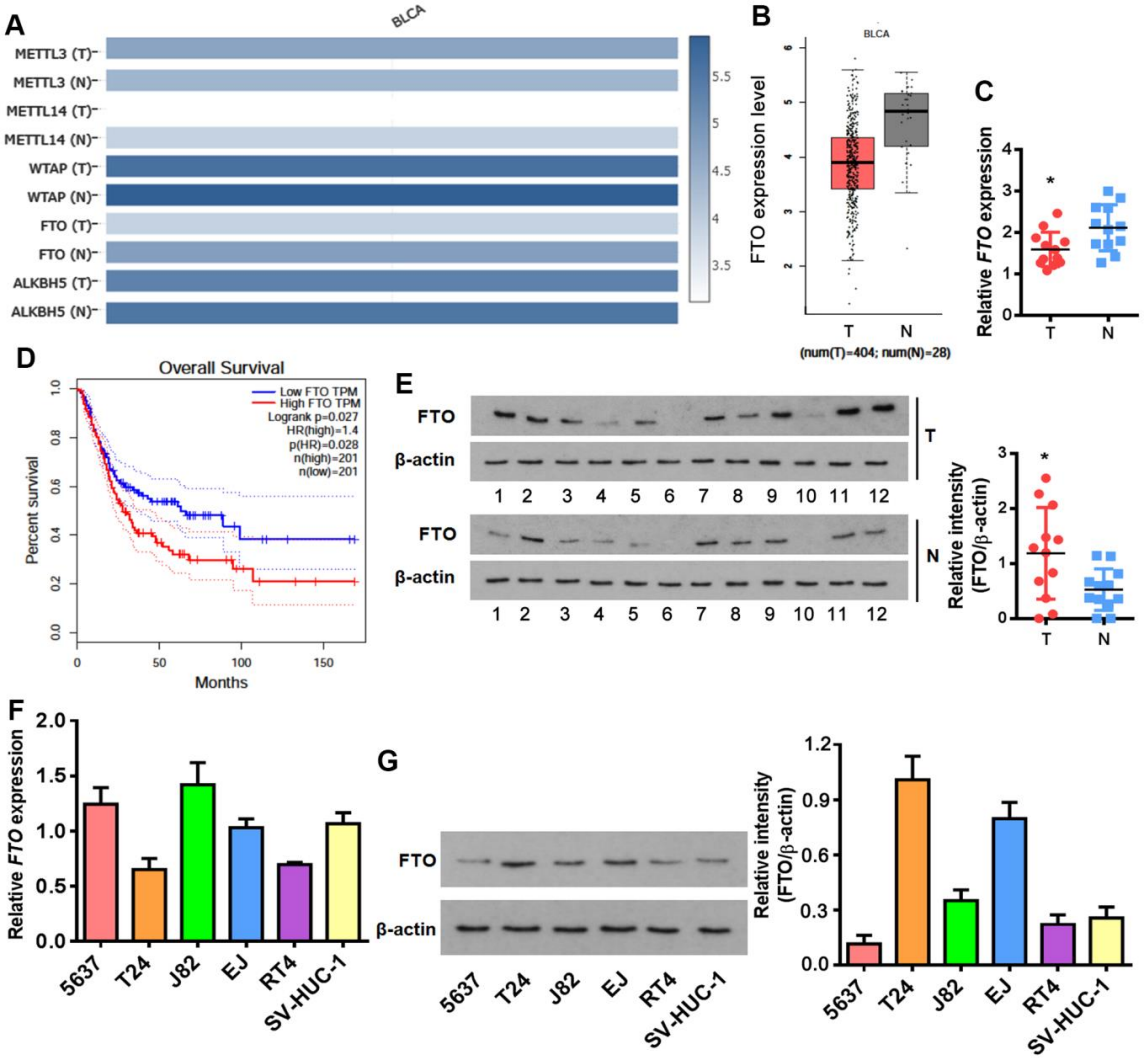
**Enrichment of FTO protein but not mRNA in BLCA.** (**A**) Heatmap showing expression of the major m6A methylases and demethylases in BLCA (T) and normal tissues (N). (**B**) Comparison of *FTO* mRNA levels between BLCA tumors (red box) and normal tissues (grey box). (**C**) Overall survival in BLCA patients with the higher FTO level was shorter as demonstrated by Kaplan-Meier analysis. (**D**) *FTO* transcript in patient-derived bladder tumor (T) and normal tissues (N) by qPCR. * p < 0.05, student’s *t*-test. (**E**) FTO protein expression in patient-derived normal tissues (N) and BLCA tumor (T) by western blot. * p < 0.05, student’s *t*-test. (**F**) *FTO* mRNA expression in BLCA and normal uroepithelial SV-HUC-1 cells by qPCR. (**G**) FTO protein profiling in BLCA and normal uroepithelial cell lines by western blot.

M6A formation is a dynamic modification under control of two classes of regulators. Methyltransferase complex (MTC) acts as a writer to install m6A, while demethylase removes m6A like an eraser [21]. None of the key writer and eraser genes were changed at mRNA level between normal and BLCA tissues except for FTO and METTL14 which were decreased in BLCA tissues (Figure 1A, 1B). Unexpectedly, FTO was negatively correlated with the survival rate of BLCA patients (Figure 1D), which is seemingly a paradox in terms of its reduced transcription in bladder tumors. We then examined FTO expression in patient-derived BLCA tissues. Interestingly, although *FTO* mRNA was lower (Figure 1C), more protein was detected in BLCA than in para-BLCA tissues (Figure 1E). The higher amounts of FTO protein but not mRNA was also detected in a panel of bladder cancer cell lines in comparison to normal uroepithelial SV-HUC-1 cells (Figure 1F, 1G). Together, these data indicated that FTO might play an oncogenic role in bladder cancer as a N^6^-methyladenosine RNA demethylase.

### USP-18 promotes FTO protein stability by inhibiting proteasomal degradation

**Figure 2:**
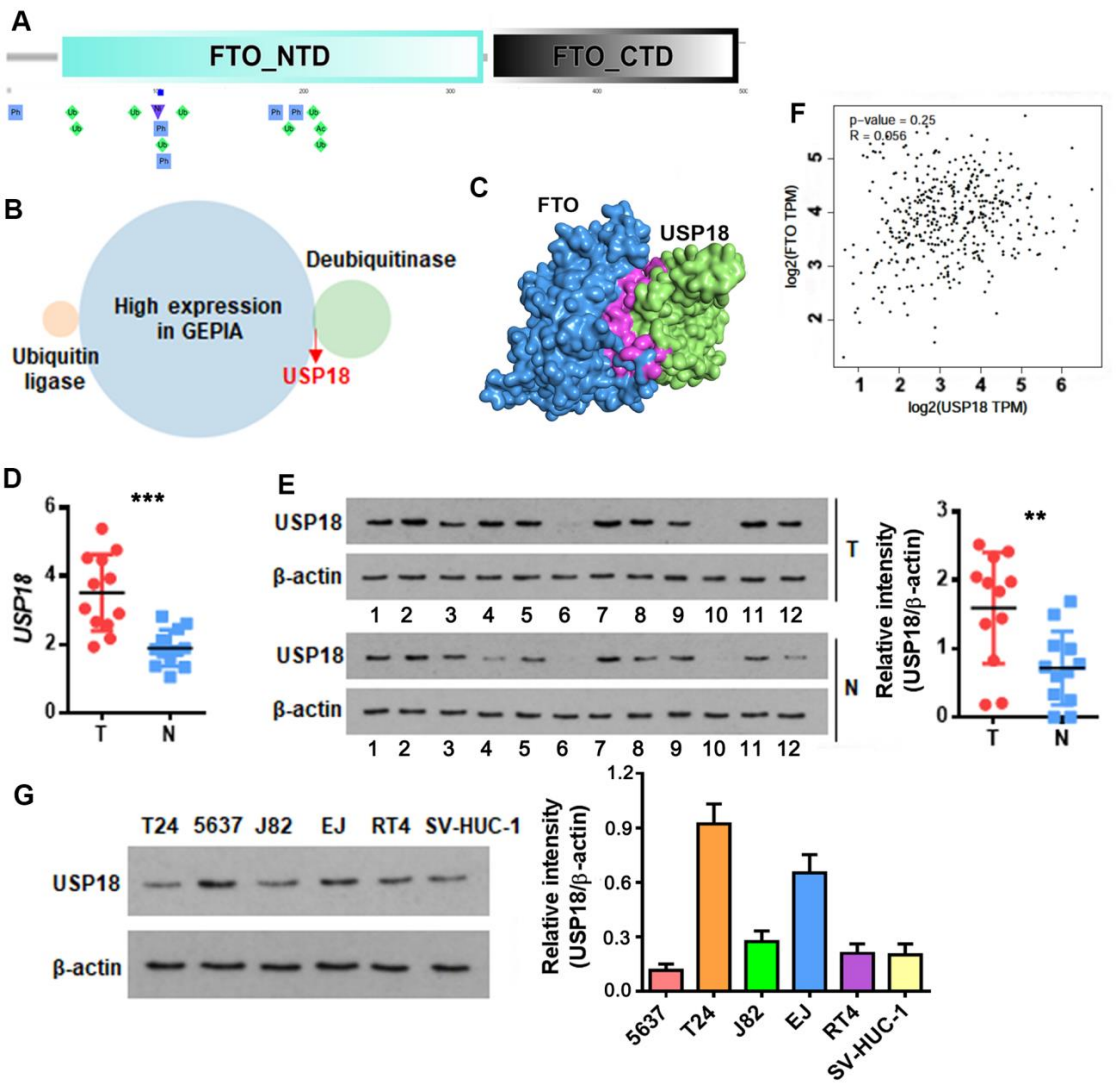
**USP18 is up-regulated in bladder cancer and potentially associated with FTO protein level.** (**A**) Schematic of the putative ubiquitination sites of K194, K211 and K216 (rhombus in green) in the N-terminal domain (NTD) of FTO as predicted by PTMcode tool. (**B**) Venn diagram showing FTO-targeting ubiquitin ligases and deubiquitinases that are significantly upregulated in BLCA. (**C**) Diagram showing the physical interaction between FTO and USP18 as predicated by HDOCK server. (**D**) *USP18* transcript in patient-derived bladder tumor (T) versus normal tissues (N). *** p < 0.001, student’s *t*-test. (**E**) USP18 protein in BLCA tumor (T) versus normal tissues (N) as determined by western blot assay. ** p < 0.01, student’s *t*-test. (**F**) Pearson correlation analysis of *FTO* with *USP18* expression. (**G**) USP18 protein expression profiling in the representative BLCA cell lines.

An increase in protein but not mRNA level was reminiscent of post-translational modification of FTO in bladder tumors. Recently, Zhu et al. reported that cellular FTO protein turnover is regulated by post-translational ubiquitination on lysine-216 [22]. Prediction through PTMcode database [23] showed three potential ubiquitination sites including K194, K211 and K216 in the N-terminal domain of FTO (Figure 2A). We further searched in UbiBrowser [24] for FTO-targeting ubiquitin ligase and deubiquitinase genes, and overlapped them separately with the upregulated BLCA signatures in Gene Expression Profiling Interactive Analysis (GEPIA) database [25]. No FTO-targeting ubiquitin ligase was upregulated in BLCA tissues (Figure 2B), while deubiquitinase USP18 was highly induced (Figure 2B). HDOCK server [26]-based protein-protein interaction analysis indicated that USP18 directly binds to FTO protein domain ranging from 190 to 220 amino acid, where the 3 putative ubiquitination sites are located (Figure 2C). Up-regulation of USP18 was further confirmed in patient-derived bladder tumors in this study (Figure 2D, 2E). However, there was no appreciable correlation between the expression of *USP18* and *FTO* at mRNA level (Figure 2F). These data implied an interaction between USP18 and FTO at protein but not mRNA level. Consistent with these patient tumor-derived data, USP18 was also up-regulated in numerous bladder cancer cell lines (Figure 2G).

**Figure 3:**
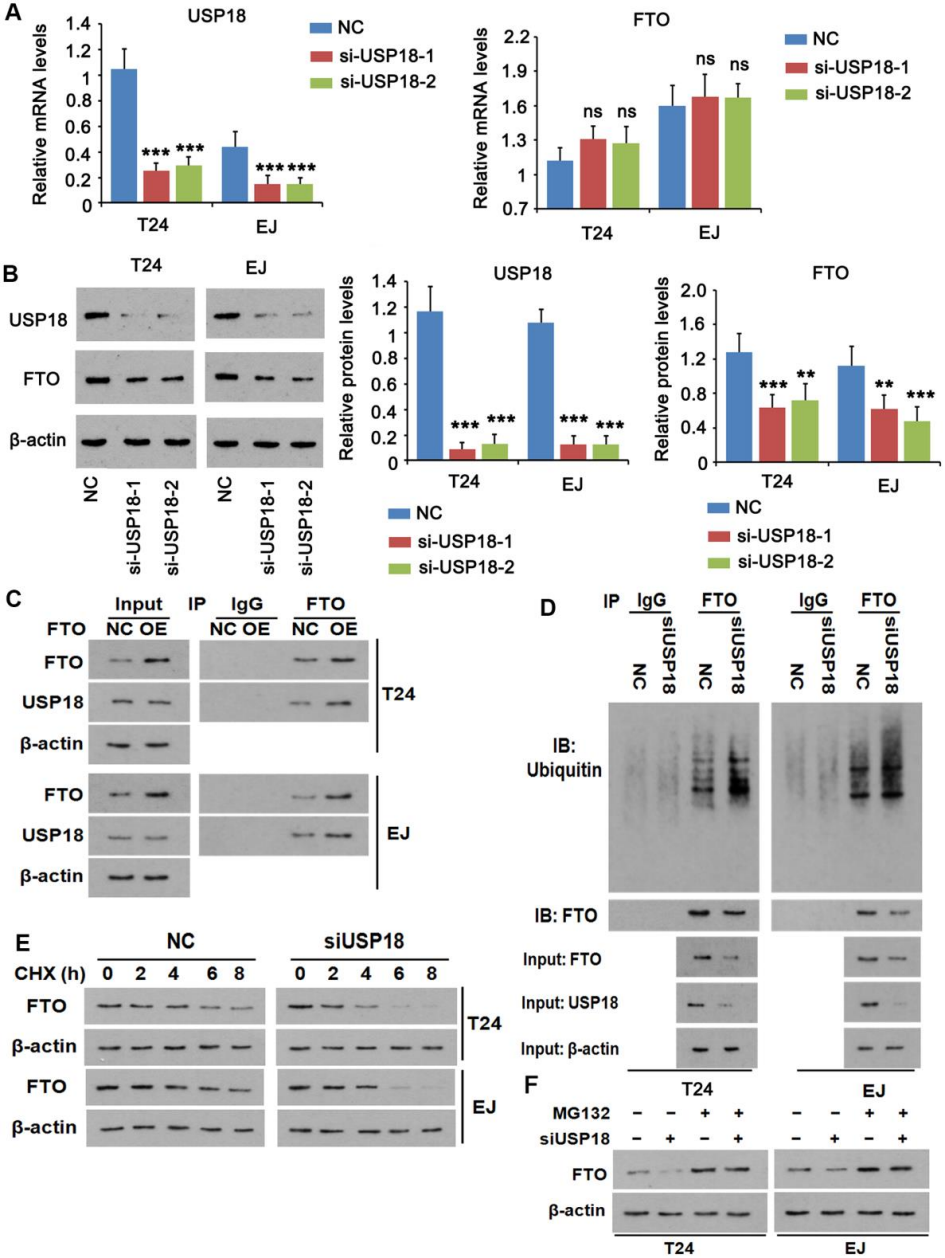
**USP18 imposes post-translational deubiquitination on FTO.** (**A**, **B**) Determining USP18, FTO mRNA (A) and protein (**B**) expression upon USP18 depletion. USP18 was knocked down by transfecting USP18 siRNA-248 (si-USP-1) and USP18 siRNA-581 (si-USP-2). NC, negative control transfected with scramble shRNA. *** p < 0.001, student’s *t*-test. n.s., non-significant. (**C**) Co-immunoprecipitation determining the direct interaction between FTO and USP18. NC, negative control; OE, overexpression. (**D**) Co-immunoprecipitation of FTO protein and ubiquitin in the presence or absence of USP18. USP18 was knocked down by transfecting USP18 siRNA-248. (**E**) Western blot determining FTO protein stability in the presence or absence of USP18. USP18 was knocked down by transfecting USP18 siRNA-248. (**F**) Western blot showing that blockage of proteasomal degradation with MG132 stabilized FTO protein upon depletion of USP18. USP18 was knocked down by transfecting USP18 siRNA-248.

For loss-of-function experiment, we depleted USP18 using two independent small interfering RNAs (siRNAs) (Figure 3A), which reduced FTO protein (Figure 3B), but not FTO mRNA in both T24 and EJ cell lines. The putative interaction was testified by co-immunoprecipitation of the two endogenous proteins (Figure 3C). Meanwhile, overexpress FTO increased the yield of the immunoprecipitated USP18 without changing its expression, indicating the strong interaction between the two proteins. Conforming to its deubiquitinase property, the physical interaction of USP18 with FTO led to a lesser extent of ubiquitination in FTO than that was appeared upon silencing of USP18 (Figure 3E). As a consequence of deubiquitination, FTO protein stability was decreased, as evidenced by an accelerated protein degradation in the absence of USP18 (Figure 3D). This was ascribed to a reinforced proteasomal protein degradation as addition of the proteasome inhibitor MG132 prevented FTO protein loss upon depletion of USP18 (Figure 3F). Taken together, increase of FTO protein in BLCA tissue was due partially to its escape from proteasomal protein degradation upon excessive USP-18-imposed deubiquitination.

### USP18 and FTO promote BLCA cell proliferation and migration

**Figure 4:**
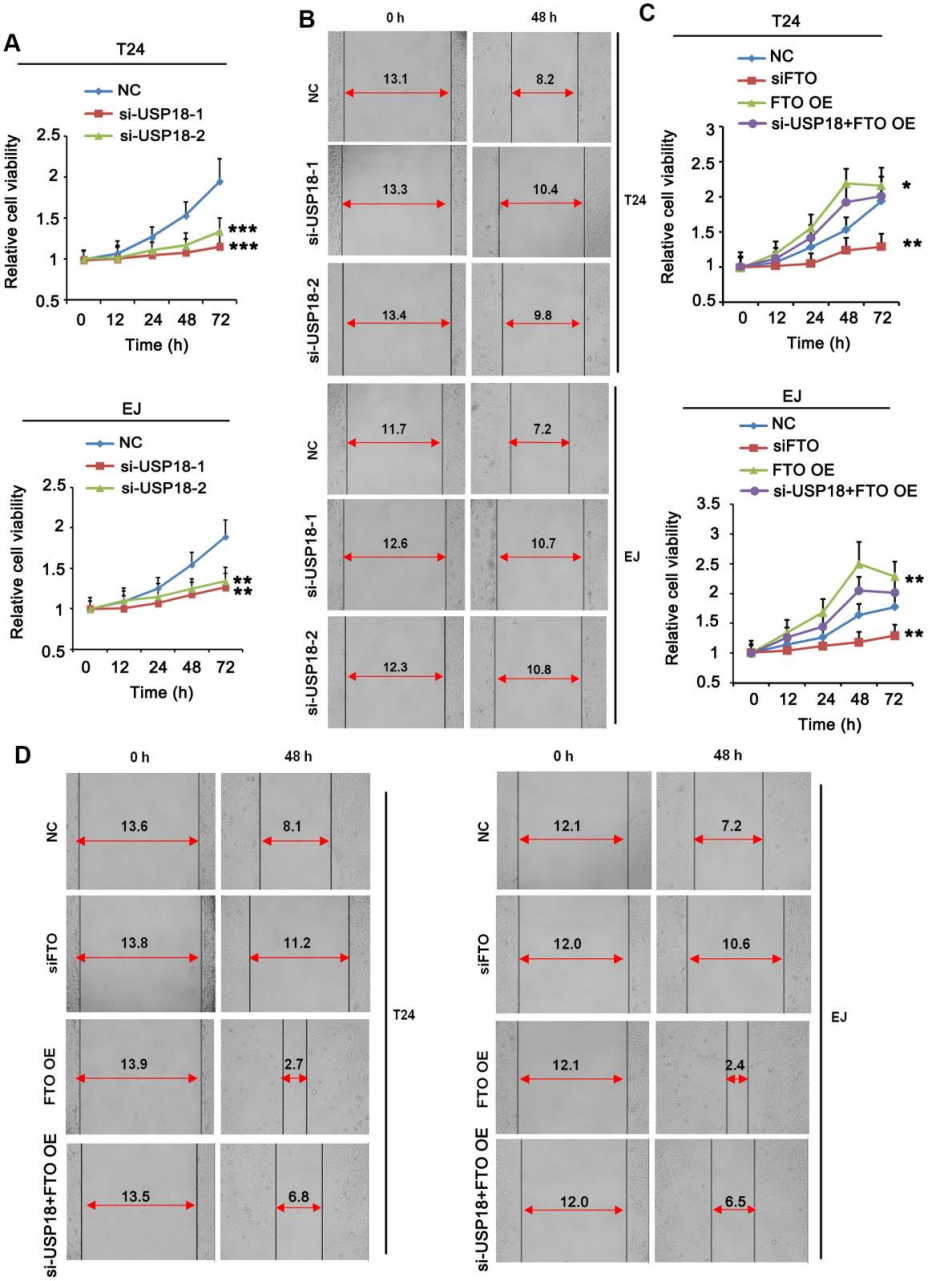
**Turn down USP18 or FTO inhibits BLCA cell proliferation and migration.** (**A**) Time-dependent BLCA cell proliferation upon depletion of USP18 by MTT assay. USP18 was knocked down by transfecting USP18 siRNA-248 (si-USP-1) and USP18 siRNA-581 (si-USP-2). ** p < 0.01, *** p < 0.001 vs. control, student’s *t*-test. (**B**) Wound-healing assay assessing the role of USP18 in BLCA cell migration. USP18 was knocked down by transfecting USP18 siRNA-248 (si-USP-1) and USP18 siRNA-581 (si-USP-2). (**C**) MTT assay assessing time-dependent BLCA cell proliferation upon depletion or expression of FTO, or depletion of USP18 together with FTO overexpression. *p < 0.05, **p < 0.01 vs. control, student’s *t*-test. (**D**) Wound-healing assay assessing BLCA cell migration upon depletion or expression of FTO, or depletion of USP18 together with FTO overexpression.

Next, we determined the roles of FTO and USP18 in the tumorigenic propensities of BLCA cells. Both USP18-depleted T24 and EJ cells displayed an apparent growth retardation (Figure 4A) as well as impaired cell migration (Figure 4B) compared to their corresponding parental cells. The phenotypes were photocopied upon silencing FTO (Figure 4C, 4D). Instead, overexpress FTO promoted proliferation (Figure 4C) and migration (Figure 4D) of BLCA cells comparing to the respective control cells. However, USP18 silencing blocked the proliferation and migration induced by FTO. These data indicated that USP18-mediated increase of FTO protein stability exacerbates BLCA progression by enhancing carcinogenic properties of the tumor cells.

### FTO stabilizes *PYCR1* mRNA by reducing its m6A methylation

**Figure 5:**
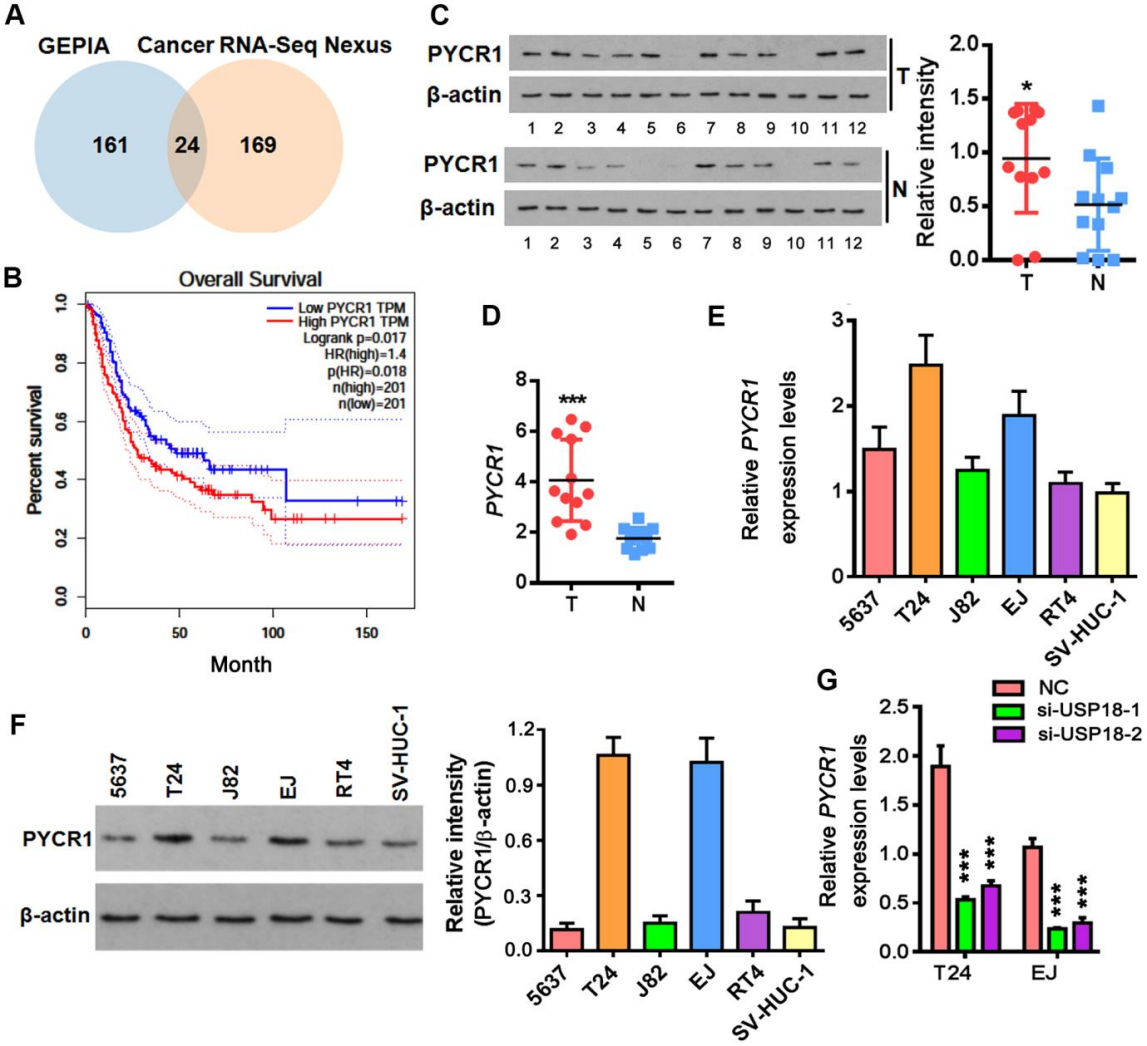
**Up-regulation of PYCR1 in bladder cancer.** (**A**) Venn diagram showing commonly upregulated BLCA signatures in both GEPIA and Cancer RNA-seq Nexus databases. (**B**) Overall survival in BLCA patients with the higher PYCR1 level was shorter as demonstrated by Kaplan-Meier analysis. (**C**, **D**) Determining PYCR1 mRNA (**C**) and protein (**D**) in patient-derived bladder tumor (T) versus normal tissues (N). * p < 0.05, *** p < 0.001, student’s *t*-test. (**E**, **F**) PYCR1 protein (**E**) and mRNA (**F**) expression in the representative BLCA cells lines. (**G**) *PYCR1* mRNA level upon USP18 depletion by qPCR. *** p < 0.001, student’s *t*-test.

**Figure 6:**
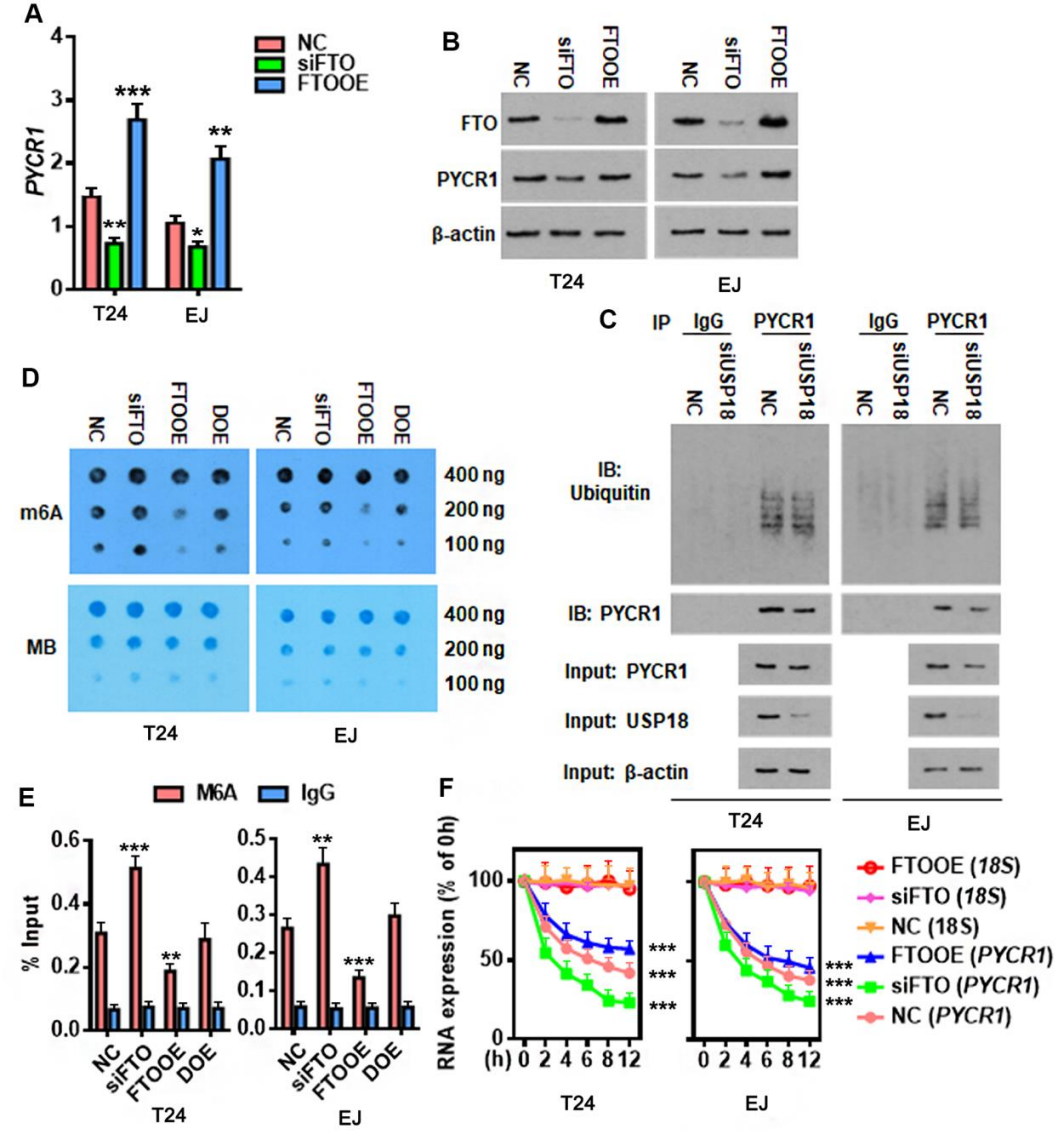
**FTO reduces m6A methylation on *PYCR1* and enhances its RNA stability.** (**A**, **B**) Determining PYCR1 mRNA (**A**) and protein (**B**) expression upon gain (FTOOE) or loss (siFTO) of FTO. * p < 0.05, ** p < 0.01, *** p < 0.001 versus NC group, student’s *t*-test. (**C**) Co-immunoprecipitation of PYCR1 protein and ubiquitin in the presence or absence of USP18. (**D**) M6A dot blot (upper) and methylene blue staining (lower) assays determining effects of FTO in the total RNA m6A contents in BLCA cells. DOE, FTO and METLL3 double overexpression. MB, methylene blue. (**E**) M6A-IP-qPCR assay determining the effects of FTO in *PYCR1* m6A methylation. DOE, FTO and METLL3 double overexpression. (**F**) qPCR determining *PYCR1* RNA stability upon gain or loss of FTO in BLCA cells. *18S* RNA was used as a normalization control.

As a major m6A demethylase, FTO stabilizes RNA by removing m6A RNA methylation. We found 24 common BLCA signatures that are upregulated in both GEPIA and Cancer RNA-seq Nexus [27] databases (Figure 5A). GEPIA overall survival analysis demonstrated that 3 out of 24 upregulated genes were negatively associated with the overall survival rate of BLCA patients (Figure 5B). Compared to the well-studied candidate genes like *KRT14* and *MMP9*, little was known about *PYCR1* in BLCA. Moreover, differential expression profiling in Cancer RNA-seq Nexus showed that *PYCR1* is significantly increased in Grade 2, 3 and 4 but not Grade 1 bladder tumors in comparison to normal tissues (Table 1), indicating its involvement in BLCA progression, invasion and metastasis. We confirmed by western blot and qPCR assays that both protein and mRNA of PYCR1 were increased in patient-derived BLCA tissues (Figure 5C, 5D) and cells lines (Figure 5E, 5F) relative to their respective controls. *PYCR1* expression was decreased upon silencing either USP18 (Figure 5G) or FTO (Figure 6A). In contrast, forced FTO expression greatly increased the amount of both mRNA and protein of PYCR1 (Figure 6A, 6B). Regarding the mechanism, we excluded the possibility of the direct deubiquitination of USP18 on PYCR1 because the amount of ubiquitinated PYCR1 remained unchanged regardless of USP18 expression (Figure 6C). It was predicted by m6Avar [28] that a few highly scored m6A-methylation sites in human *PYCR1* orthologous gene (Table 2). Indeed, knockdown of FTO increased the global mRNA m6A level in both T24 and EJ cells, while the opposite was true when overexpressing FTO, which could be rescued by forced expression of N6-adenosine-methyltransferase catalytic subunit METTL3 (Figure 6D). Specifically, MeRIP-qPCR results demonstrated that m6A level on *PYCR1* was increased upon depletion of FTO (Figure 6E). Instead, overexpress FTO decreased *PYCR1* m6A level and co-express METTL3 restored it to a level comparative to the control cells. Furthermore, mRNA stability assay showed that loss of FTO accelerated the degradation of *PYCR1* mRNA, whereas gain of FTO extended its half-life indicating an increase in mRNA stability (Figure 6F). Aa a whole, these data reveal a gene regulation signaling cascade via USP18/FTO axis in BLCA.

**Table 1:** Expression of PYCR1 at different stages of BLCA.

**BLCA stage**	**Name**	**EXP1**	**EXP2**	**log2**	**adjusted P-value**
Grade 2	PYCR1	36.50778	6.54696979	2.479304563	1.05E-05
Grade 3	PYCR1	46.8357	6.54696979	2.83870934	4.00E-07
Grade 4	PYCR1	56.19453	6.54696979	3.101530491	9.26E-08

**Table 2:** Highly-scored m6A modification sites in PYCR1 mRNA.

**m6A**	**m6A source**	**Species**	**Gene**	**Database**	**Transcriptome regulation**
chr17:79890560(-)	miCLIP (High)	Human	PYCR1	dbSNP147	RBP 2
chr17:79891172(-)	MeRIP-Seq (Medium)	Human	PYCR1	dbSNP147	RBP 3 Splice 1
chr17:79891187(-)	MeRIP-Seq (Medium)	Human	PYCR1	dbSNP147	RBP 3 Splice 2
chr17:79891187(-)	MeRIP-Seq (Medium)	Human	PYCR1	dbSNP147	RBP 3 Splice 2

### PYCR1 restores FTO-depleted BLCA cell proliferation and migration *in vitro*

**Figure 7:**
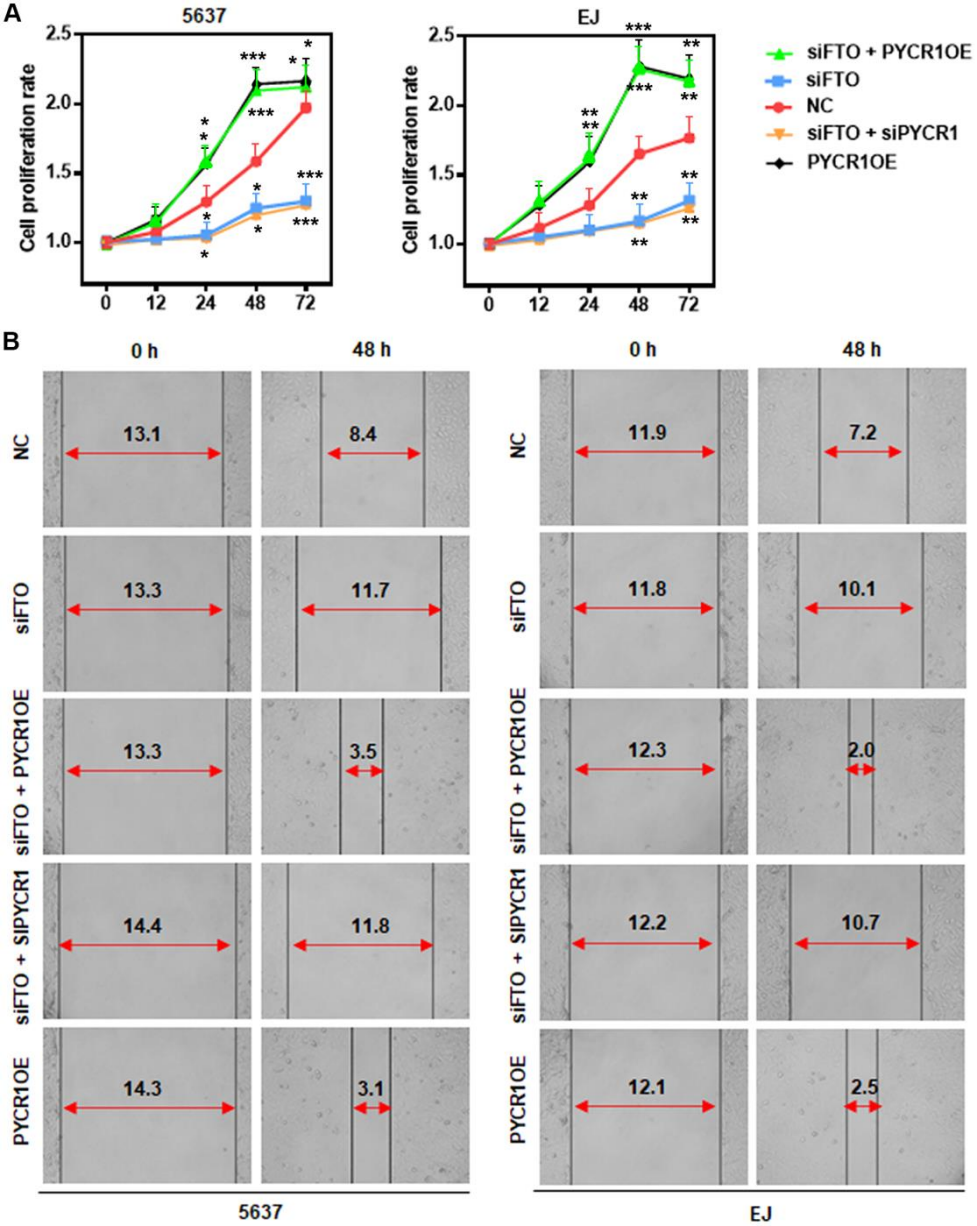
**PYCR1 restores FTO-depleted BLCA cell defects in proliferation and migration.** (**A**) MTT assay determining the role of PYCR1 in BLCA cell proliferation. (**B**) Wound-healing assay assessing the effect of PYCR1 in BLCA cell migration.

Our results showed that FTO promoted BLCA cell proliferation and migration (Figure 4C, 4D). We hypothesized that it might be mediated by PYCR1 because ectopic PYCR1 expression facilitated tumor cell proliferation (Figure 7A) and migration (Figure 7B). Indeed, forced expression of PYCR1 restored the propagation and invasion defects of FTO-depleted BLCA cells (Figure 7A, 7B), indicating the essential tumor-promoting role of USP18/FTO/PYCR1 signaling network in BLCA.

### PYCR1 rescues xenograft tumor growth defect upon loss of FTO

**Figure 8:**
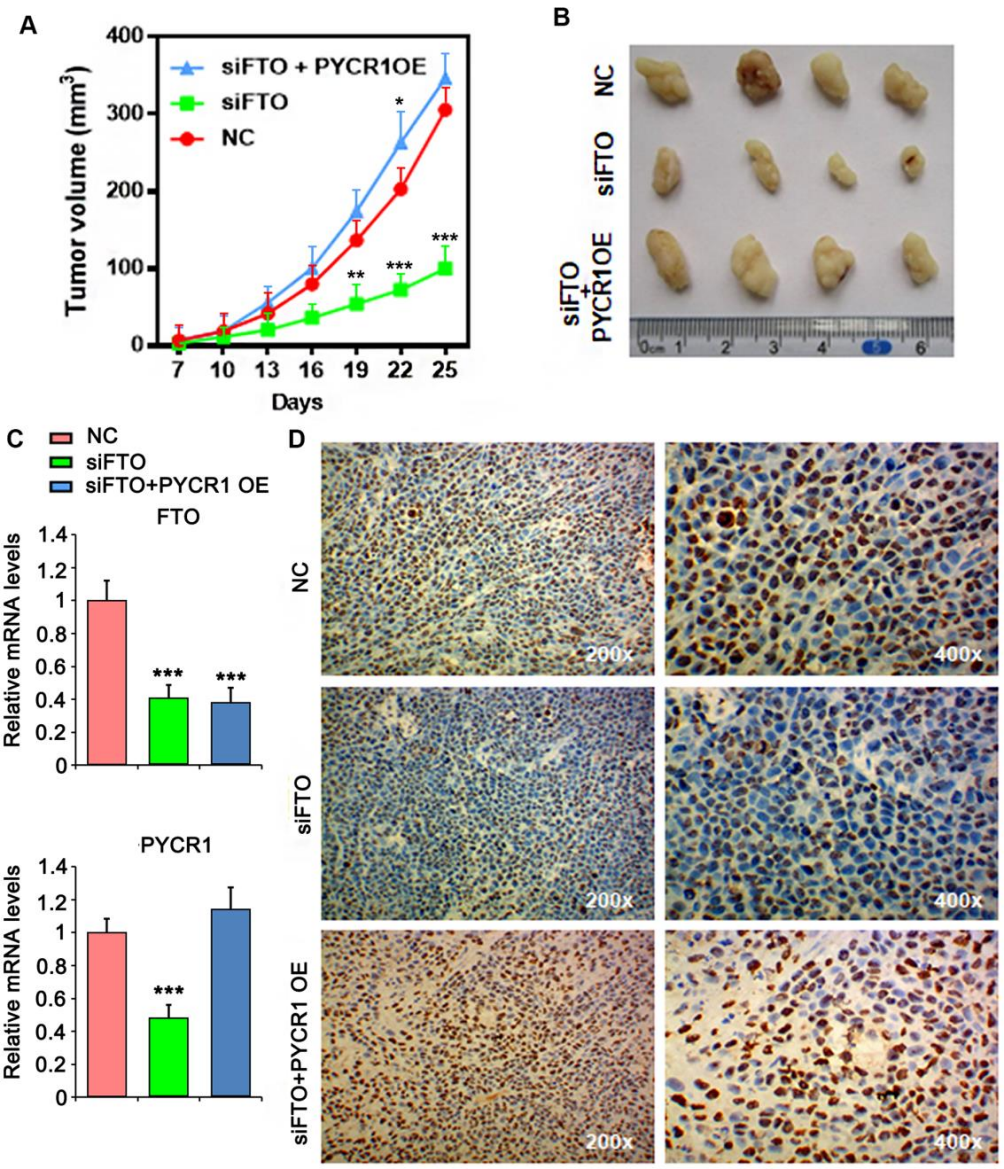
**PYCR1 promotes FTO-deficient xenograft tumor growth.** (**A**) Tumor growth curves as recorded by measuring tumor volume. (**B**) Xenograft tumor morphology. (**C**) qPCR determining FTO and PYCR1 expression in xenograft tumor. (**D**) Ki67 immunohistochemistry on xenograft tumor sections. NC, negative control. OE, overexpression.

Finally, we assessed the roles of FTO and PYCR1 in xenograft tumor growth *in vivo*. As expected, loss of FTO significantly compromised xenograft tumor growth in nude mice compared to the control group (Figure 8A, 8B, 8C). However, simultaneously overexpress PYCR1 promoted FTO-depleted tumor growth (Figure 8A, 8B, 8C). Consistently, immunohistochemical staining on these tumors displayed that comparing to the control tumors, cells in FTO-deficient tumors were less proliferative as indicated by the sporadic Ki67-positive staining (Figure 8D). Instead, FTO-depleted but PYCR1-overexpressing tumors were filled with Ki67-positive proliferating cells (Figure 8D). Taken together, these data suggest that FTO/PYCR1 axis promotes bladder tumorigenesis *in vivo*.

## DISCUSSION

Functional studies on RNA m6A methylation have thrived since the identification of the first RNA demethylase FTO, which revolutionarily revealed that RNA methylation is a dynamic and reversible process [2]. Emerging evidence indicate that changes in these m6A-regulating proteins promote cancer cell proliferation, survival, tumor initiation and progression by inducing the expression of key oncogenic genes [29]. Our current study highlighted FTO as a potential marker predicting a poor prognosis of BLCA and demonstrated that FTO increased *PYCR1* mRNA stability through its m6A demethylase activity to facilitate BLCA development.

*FTO* is highly expressed in many types of cancer such as acute myeloid leukemia with t(11q23)/MLL rearrangements, t(15;17)/PML-RARA fusion, FLT3-ITD or NPM1 mutation [18], human cervical squamous cell carcinoma tissue [30] and gastric cancer [31] *etc*. Nevertheless, it is decreased in BLCA tumors compared to normal tissues. This is seemingly contradictory to its negative correlation with the overall survival of BLCA patients. We demonstrated that FTO protein is however, highly expressed in tumor tissues and cells due to the upregulation of USP18. It is well-established that proteins are subject to ubiquitin-proteasome-mediated degradation upon conjugation of ubiquitin maker [32]. Deubiquitinating enzymes catalyze removal of ubiquitin markers from target proteins and thus prevent them from proteasomal degradation. The activity of these enzymes influences proteins turnover rate, stability and subcellular localization, leading to changes in cell homeostasis and propensity [33]. Indeed, many deubiquitinases are considered as oncogenes or cancer suppressor genes based on their downstream target proteins. Targeting deubiquitinases is thus a promising therapeutic option against cancer [34]. Our results indicate the development of pharmacological inhibitor to USP18 is promising in the future BLCA treatment.

FTO was initially identified because of the strong association of multiple SNPs in its first intron with type 2 diabetes [35]. However, the underlying mechanism remained unknown until the discovery of FTO as an m6A demethylase, which accounts for most of its functions in controlling body weight, obesity and adipogenesis [36–39]. Generally, m6A deposition decreases the expression of methylated mRNAs and shortens their half-lives, while loss of m6A boosts gene transcription and increases RNA stability [40]. Global m6A mapping studies revealed that m6A sites are enriched in genes regulating development and cell fate specification [6, 41, 42]. Instead, little m6A modification was found in highly stable transcripts like housekeeping genes [43]. PYCR1 encodes an enzyme that catalyzes the NAD(P)H-dependent conversion of pyrroline-5-carboxylate to proline [44], and thus is critical for cellular nucleotide metabolism. In this study, we demonstrated that *PYCR1* is highly expressed in BLCA tissue and cells, and negatively associated with the overall survival rate, indicating its oncogenic function in BLCA. PYCR1 was also reported to promote proliferation and inhibit apoptosis in non-small cell lung cancer [45]. In contrast, *PYCR1* interference inhibits cell growth and survival in hepatocellular cancer [46]. Of note, our work reveals the abnormal *PYCR1* expression in BLCA tissue as a consequence of FTO-induced m6A RNA demethylation, providing the potential mechanism accounting for the dysregulation of *PYCR1* in other types of human cancer as well.

In conclusion, we showed in the current study the abnormal USP18/FTO/PYCR1 signaling network in BLCA tissue, which favors cell proliferation and migration *in vitro* as well as tumor initiation and progression *in vivo*. Our work not only provides a novel mechanistic insight into BLCA development but also offers new targets for BLCA diagnosis, prognosis and therapy.

## MATERIALS AND METHODS

### Tissue collection and ethical statement

Bladder urothelial tumor samples and the adjacent normal tissues (para-BLCA) were collected from 12 patients following radical cystectomy in the Hunan Provincial People’s Hospital. All histologic diagnoses were conducted by the pathology department of Hunan Provincial People’s Hospital. Informed consents were signed by all the patients involved. This study was permitted by the Ethics Committee at the Hunan Provincial People’s Hospital.

### Cell lines and treatment

BLCA cell lines including 5637, T24, J82, EJ and RT4 as well as the normal uroepithelial cell line SV-HUC-1 were purchased from the Chinese Academy of Sciences Cell Bank (Shanghai, China). Base media used in cell culture included RPMI-1640 for 5637 and EJ, McCoy's 5a Medium Modified for T24 and RT4, Eagle's Minimum Essential Medium for J82 as well as F12K for SV-HUC-1 cells. The complete growth media were made up with the corresponding base medium supplemented with 10% fetal bovine serum (FBS) (Sigma-Aldrich, St. Louis, MO, USA). For FTO protein stability study, T24 and EJ cells were treated with 20 mg/ml cycloheximide (CHX) (Millipore Sigma, #C7698) or 5 μM MG132 (Sigma-Aldrich, #1211877-36-9) for the indicated time, and then harvested for protein extraction and western blot analysis.

### Transfection and infection for overexpression or knockdown

5 x 10^4^ T24 or EJ cells were seeded in 6-well plate on day before transfection, and transfected with each siRNA using Lipofectamine^TM^ 2000 (Invitrogen; Thermo Fisher Scientific, Inc., USA) by following the instruction of manufacture. Sequences of the siRNA constructs were as follows: USP18 siRNA-248 (sense: 5′-UCAUGUUGCUGUCUUCUUCTT-3′; antisense: 5’-GAAGAAGACAGCAACAUGATT-3’), USP18 siRNA-581 (sense: 5′-AGAGUUUGAGGUACAGUUGTT-3′; antisense: 5’-CAACUGUACCUCAAACUCUTT-3’), FTO siRNA (sense: 5′-AAAUAGCCGCUGCUUGUGATT-3′; antisense: 5’-UCACAAGCAGCGGCUAUUUTT-3’) [47], Negative control FAM (sense: 5′-UUCUCCGAACGUGUCACGUTT-3′; antisense: 5’-ACGUGACACGUUCGGAGAATT-3’).

For *in vivo* xenograft experiment, lentiviral vectors expressing shFTO, PYCR1 and the empty lentiviral vector were purchased from GeneChem (Shanghai, China). Stably infected T24 cells were selected by puromycin (2 μg/ml).

### Bioinformatics

The overall survival and gene expression analysis of the major m6A methylases and demethylases were performed based on the Signature-based statistics tool in GEPIA 2 (http://gepia2.cancer-pku.cn/#index). Protein ubiquitination sites were predicted through the online tools provided by PTMcode 2 (https://ptmcode.embl.de/index.cgi). The USP18 and FTO interaction model was predicted using protein-protein docking analysis tool in HDOCK Server (http://hdock.phys.hust.edu.cn). For searching the commonly upregulated signatures in BLCA, the cutoff threshold of log2 fold change was 2.0 in GEPIA and 3.0 in Cancer RNA-Seq Nexus, respectively. The potential m6A methylation sites in PYCR1 RNA was predicated by m6Avar (http://m6avar.renlab.org).

### RNA extraction and quantitative PCR (qPCR)

Total RNA from BLCA tissue and cells were extracted using TRIzol reagent (Invitrogen; Thermo Fisher Scientific, Inc., USA), and reverse-transcribed using RevertAidTM H Minus First Strand cDNA Synthesis Kit (Fermentas). qPCR was then performed using SYBR Green PCR Master Mix (ABI 4309155) in ABI7900 Realtime-PCR machine. The primers used were β-actin (forward: 5’- CATGTACGTTGCTATCCAGGC-3’; reverse: 5’-CTCCTTAATGTCACGCACGAT-3’), FTO (forward: 5’-AGCAGAGCAGCATACAACGT -3’; reverse: 5’-TCCCTGCCTTCGAGATGAGA -3’), USP18 (forward: 5’-CCTGAGGCAAATCTGTCAGTC-3’; reverse: 5’-CGAACACCTGAATCAAGGAGTTA-3’), and PYCR1 (forward: 5’- AAGATGCTGCTGCACTCAGA-3’; reverse: 5’-CACCTTGTCCAGGATGGTCT-3’).

### Western blot

BLCA tissues or cells were lysed using RIPA buffer (1% NP-40, 0.1% SDS, 50 mM DTT) supplemented with protease inhibitor cocktail containing 2 μg/ml Aprotinin, 2 μg/ml Leupeptin and 1 mM PMSF. The lysates were subject to ultrasonication followed with centrifuged for 10 min at 9,000 rpm to collect the supernatants. Equal amount of total proteins from each sample were resolved by 10% SDS-PAGE and electroblotted onto polyvinylidene difluoride membranes. Immunoblotting was performed with anti-FTO (RD, #MAB7208), anti-USP18 (Abcam, #ab168478), anti-PYCR1 (Abcam, #ab226340) and anti-β-actin (Ptgcn, #66009-1-Ig) antibodies.

### Co-immunoprecipitation (Co-IP)

BLCA cells grown in 10-cm culture dishes were lysed with RIPA buffer. Human IgG (Bioss, #bs-0297P, 1:150), anti-FTO antibody (RD, #MAB7208, 1:50) or anti-PYCR1 antibody (Abcam, #ab226340, 1:100) were incubated separately with each cell lysate overnight at 4° C with gentle rotation. 20 μl Protein A/G agarose beads (Beyotime Biotechnology, #P1012) were added and incubated at 4° C with gentle rotation for 2 h. After the sequential wash with PBS and cell lysis buffer, the agarose beads were resuspended in 20 μl 1x SDS-PAGE loading buffer, and boiled for western blot analysis on Ubiquitin (Proteintech, 10201-2-AP, 1:200) or USP18 (Abcam, #ab168478, 1:500).

### Cell viability assay

BLCA cells were seeded into 96-well plates (1 x 10^4^ cells/well), cell viability was measured at 0, 12, 24, 48 and 72 h after seeding using MTT cell proliferation and Cytotoxicity Detection Kit (KeyGEN Biotech, #KGA312) by following the instruction of manufacture.

### Wound-healing assay

BLCA cells were grown in 24-well plates till formation of a confluent monolayer which was then wounded through scratching with a sterile 200 μl pipette tip, and proceeded with culturing in the medium supplemented with 1% FBS for 48 hours. The wound areas were recorded at the starting (0-hour) and ending (48-hour) timepoints, respectively, using a digital camera system. The migration distance was measured by ImageJ software (https://imagej.nih.gov/ij/).

### Global m6A dot blotting

Gradient diluted total RNAs were spot loaded and UV crosslinked onto a nylon membrane (GE Healthcare). The membrane was then blocked with 5% nonfat milk in PBST for 1 h and incubated with an anti-m6A antibody (Synaptic Systems, #202111) overnight at 4° C. The horseradish peroxidase conjugated secondary antibody was subsequently added, and incubated for 1 h at room temperature. The membrane was washed off the unbound secondary antibody and developed with Amersham ECL Prime Western Blotting Detection Reagent (GE Healthcare, #RPN2232). The same membrane was proceeded with a staining using 0.02% methylene blue in 0.3 M sodium acetate (pH 5.2) for 2 h followed by a rinse with water for 5 h.

### Methylated RNA immunoprecipitation (MeRIP)-qPCR

MeRIP was performed using EZ-Magna RIP^TM^ RNA-Binding Protein Immunoprecipitation Kit (Millipore, #17-701) by following the manual instruction. The enriched m6A methylated RNA was reverse-transcribed and quantified with *PYCR1* expression by qPCR.

### RNA stability assay

T24 or EJ cells treated with 5 μg/ml Actinomycin D were harvested at indicated timepoints for total RNA extraction and qPCR analysis. 18S RNA (forward forward: 5’-CAGCCACCCGAGATTGAGCA-3’; reverse: 5’-TAGTAGCGACGGGCGGTGTG-3’) was used as the internal control.

### *In vivo* xenograft experiments

Male BALB/c nude mice (4-6-week-old, n = 4 per group) were purchased from Beijing HFK Bioscience Co. Ltd (Beijing, China) and maintained under pathogen-free conditions. T24 cells were subcutaneously injected into the mice (1 x 10^6^ cells / injecting site). Tumor size was evaluated around 1 week after the indicated time points based on the formula of Volume = a x b^2^ x 0.52 (a, long diameter; b, short diameter).

### Immunohistochemistry

Xenograft tumor tissues were fixed in 10% buffered formalin for 24 h and embedded in paraffin. The deparaffinized and rehydrated sections were blocked for endogenous peroxidase by 20 min incubation in 3% hydrogen peroxide followed with antigen retrieval at 121° C in citrate buffer (10 mM, pH6.0) for 10 min. After free cooling to room temperature, the sections were blocked for non-specific binding with normal goat serum (1:10) for 30 min at room temperature, and subject to an incubation with anti-Ki67 monoclonal antibody (1:100, Dako, Glostrup, Denmark) overnight at 4° C. The next day, the sides were washed and incubated with the biotinylated secondary antibody at 37° C for 30 min, and subsequently incubated with a 1:200 streptavidin-biotin-peroxidase complex (Sigma, St. Louis) for 30 min. Reactive products were visualized with 3,3’-diaminobenzidene (DAB) as the chromogen, and nuclei were counter-stained with hematoxylin.

### Statistical analysis

Data are expressed as the mean ± standard deviation of three independent experiments. Two-tailed Student’s *t*-test was performed using GraphPad Prism 6 software. P < 0.05 was considered as significant.

### Ethics approval and consent to participate

Informed consents were signed by all the patients involved. This study was permitted by the Ethics Committee at the Hunan Provincial People’s Hospital.

### Consent for publication

Written informed consent for publication was obtained from all participants.

### Availability of data and materials

The datasets generated/analyzed in the present study are available upon reasonable request from the corresponding author.
